# TRPC6 mediates high glucose-induced mitochondrial fission through activation of CDK5 in cultured human podocytes

**DOI:** 10.3389/fphys.2022.984760

**Published:** 2022-09-21

**Authors:** Haomiao Yu, Yili Chen, Huimin Ma, Zihan Wang, Rui Zhang, Jundong Jiao

**Affiliations:** ^1^ Department of Nephrology, The Second Affiliated Hospital of Harbin Medical University, Harbin, China; ^2^ Institute of Nephrology, Harbin Medical University, Harbin, China

**Keywords:** podocyte, high glucose, mitochondrial fission, TRPC6, CDK5, calpain-1

## Abstract

Mitochondrial abnormalities contribute to the development of diabetic nephropathy (DN). However, the precise mechanisms of mitochondrial dysfunction in DN remain unclear. Transient receptor potential canonical channel-6 (TRPC6), a non-selective cation channel permeable to Ca^2+^, has been shown to regulate mitochondrial dynamics. This study was therefore aimed to explore the regulatory role and mechanisms of TRPC6 in high glucose (HG)-induced mitochondrial dysfunction in podocytes. Here we found that TRPC6 expression and TRPC6-induced Ca^2+^ influx were increased in HG-treated podocytes. Furthermore, the TRPC6 inhibitor and TRPC6 siRNA ameliorated mitochondrial dysfunction and apoptosis in HG-treated podocytes. BAPTA-AM, an intracellular calcium chelating agent, attenuated mitochondrial fission under HG conditions as well. Then, we found the activity of calpain and cyclin-dependent kinase 5 (CDK5) was markedly enhanced in HG-treated podocytes, which can be blocked by pretreatment with the TRPC6 inhibitor. Calpain-1 inhibition by calpeptin or by calpain-1 siRNA transfection not only attenuated HG-induced mitochondrial fission but also reduced the activity of CDK5. Additionally, the CDK5 inhibitor and its siRNA decreased mitochondrial fragmentation in HG-treated podocytes. Collectively, we revealed the essential role of TRPC6 in regulating HG-induced mitochondrial fission and apoptosis through the calpain-1/CDK5 pathway in human podocytes, which may provide new insights into the pathogenesis of DN.

## Introduction

Diabetic nephropathy (DN) is a common complication of diabetes, affecting millions of people around the world. Proteinuria, one of the main syndromes of DN, is caused by impairments of the glomerular filtration barrier (Maiti, 2021). In DN, proteinuria and decreased renal function are linked with podocyte damage (Dai et al., 2017; Tung et al., 2018). Since kidneys have high energy and oxygen demands, mitochondria are closely connected with renal function. A series of studies have shown that dysfunctional mitochondria in podocytes play a critical role in the development and progression of DN (Forbes and Thorburn, 2018; Gujarati et al., 2020; Su et al., 2020). However, the underlying molecular mechanism remains unclear.

Mitochondria are key organelles in podocytes preserving mitochondrial integrity and functions by balancing mitochondrial dynamics, including mitochondrial fission and fusion (Mishra and Chan, 2016). Mitochondrial dysfunction and morphological abnormalities caused by abnormal mitochondrial dynamics contribute to excessive reactive oxygen species (ROS) production and cell apoptosis, and play an important role in the pathology of DN (Forbes and Thorburn, 2018). Mitochondrial dynamics are mediated by GTPases, including dynamin-related protein 1 (Drp1), mitofusin 1 and 2 (Mfn1 and Mfn2), and optic atrophy 1 (Opa1). Drp1 is a mitochondrial outer membrane protein, which is essential for mitochondrial fission. Opa1 mediates the fusion of the mitochondrial inner membrane, and Mfn1 and Mfn2 regulate the fusion of the mitochondrial outer membrane (Tilokani et al., 2018). The activity of Drp1 is mainly mediated by phosphorylation on its serine residues. Several studies have identified that high glucose (HG) treatment induces mitochondrial fission by promoting phosphorylation of Drp1 at Serine 616 (S616) (Chen et al., 2020; Wang et al., 2021; Wu et al., 2021). Recent observations indicate that cyclin-dependent kinase 5 (CDK5) mediates podocyte mitochondrial fission by regulating Drp1-S616 phosphorylation in DN (Wang et al., 2021). The activity of CDK5 depends on co-activators, p39, p35, and p25 (the 208-residue carboxy-terminal fragment of p35). Since p25 has a long half-life and can lead to the activation and mislocalization of CDK5, CDK5/p25 complex shows stabilized structure and prolonged activity (Pao and Tsai, 2021). It has been widely reported in the nervous system that calpain upregulates the activity of CDK5 by cleaving p35 to p25 in a Ca^2+^-dependent manner, which remains to be explored in podocytes (Kusakawa et al., 2000; Park et al., 2017).

Transient receptor potential canonical channel-6 (TRPC6) is a non-selective calcium channel that plays a critical role in the pathogenesis of renal diseases. TRPC6, together with Nephrin, Podocin, and other important slit diaphragm molecules, forms a signaling complex to preserve the structural and functional integrity of the foot process and slit diaphragm in podocytes (Hall et al., 2019). Studies have shown increased TRPC6 expression and activity in diabetic models *in vivo* and *in vitro* (Staruschenko et al., 2019). For instance, HG enhances the TRPC6-dependent Ca^2+^ influx in cultured human podocytes (Yang et al., 2013). Several studies have identified that increasing intracellular Ca^2+^ triggers mitochondrial fission through the Drp1-dependent pathway in cardiac muscle cells and liver cells (Hom et al., 2007; Hom et al., 2010; Wu et al., 2021). Recent reports have revealed that TRPC6 regulates Drp1-mediated mitochondrial fission in dentate granule cells (Ko and Kang, 2017), but the effect of TRPC6 on mitochondrial dynamics in podocytes is still unknown. Adenosine monophosphate-activated protein kinase (AMPK) has been shown to mediate mitochondrial fragmentation and ROS production, by regulating Drp1 in terms of phosphorylation and translocation to mitochondria (Mao et al., 2022; Wang et al., 2022). In previous studies, TRPC6 has been reported to play a critical role in mitophagy *via* the AMPK pathway in podocytes (Rachubik et al., 2018; Rogacka, 2021). Calpain-1, one of the nonlysosomal cysteine proteases consisting of an 80 kDa isoform-specific catalytic domain, has been widely demonstrated to be activated by TRPC6-dependent Ca^2+^ influx in podocytes (Verheijden et al., 2018). The effect of calpain-1 on mitochondrial fission has been reported in cardiac diseases (Shi et al., 2021), whereas it is unclear in podocytes.

We therefore hypothesized that TRPC6-mediated Ca^2+^ influx regulates HG-induced mitochondrial fission by CDK5 activation in podocytes. In the present study, we explored the effect of TRPC6 on mitochondrial fission and the potential molecular mechanism in HG-treated podocytes. Our findings indicate that TRPC6 may regulate HG-induced mitochondrial fission through the Ca^2+^/calpain-1/CDK5 pathway in podocytes.

## Materials and methods

### Cell culture, treatment, and transfection

The conditionally immortalized human podocyte cell line was kindly provided by Dr. Moin A. Saleem (Academic Renal Unit, University of Bristol, UK). Podocytes were cultured in the medium which contained RPMI 1640 medium (Gibco, United States) with 10% fetal bovine serum (Gibco, United States), and insulin-transferrin-selenium (ITS) (Gibco, United States) at 33°C. Podocytes differentiated when cultured at 37°C for 10–14 days. The differentiated cells were treated with normal glucose (Control), 30 mM mannitol (HM) or 30 mM glucose (HG). Podocytes were respectively treated with 2-aminoethoxydiphenyl borate (2-APB, 100 μM, Sigma, United States), BAPTA-AM (10 μM, MedChemExpress, United States), roscovitine (10 μM, Sigma, United States), calpeptin (1 μM, Sigma, United States), thapsigargin (TG, 1 μM, Sigma, United States), 1-oleoyl-2-acetyl-glycerol (OAG, 100 μM, Sigma, United States) and DMSO. The X-treme GENE siRNA Transfection Reagent (Roche, Switzerland) was used to transiently transfect podocytes with TRPC6 siRNA, CDK5 siRNA, calpain-1 siRNA, or scrambled siRNA (Santa Cruz Biotechnology, United States) according to the manufacturer’s protocols. Transfected podocytes were examined within 24–48 h post-transfection.

### Real-time quantitative PCR

Total RNA from podocytes was extracted with Trizol reagent (Invitrogen, United States) and reverse transcribed with the Reverse transcription kit (Trans, China) according to the manufacturer’s instructions. qPCR was performed using FastStart Universal SYBR Green Master (Roche, Switzerland) on LightCycler 96 Instrument (Roche, Switzerland). The primers applied are as follows: TRPC6 Forward: 5′- GCCAATGAGCA TCTGGAAAT-3′, Reverse: 5′-TGGAGTCAC ATCATGGGAGA-3'; GAPDH was used as an internal reference. The relative expression levels of the target genes were calculated by 2^−ΔΔCT^ method.

### Western blotting

Western blotting was performed as described previously (Yang et al., 2013) and probed with the following primary antibodies: TRPC6 polyclonal rabbit antibodies (1:200, Alomone Labs, Israel), calpain-1 monoclonal rabbit antibodies (1:1,000, Abcam, United States), Drp1 monoclonal rabbit antibodies, p-Drp1 polyclonal rabbit antibodies, Mfn2 monoclonal rabbit antibodies, Opa1 monoclonal rabbit antibodies (1:1,000, Cell Signaling Technology, United States), or *β*-Actin rabbit antibody (1:5,000, ABclonal, China). The membranes were washed carefully and then incubated with fluorescence-conjugated goat anti-rabbit secondary antibodies (Invitrogen, United States) for 1 h. The results were quantified by the Odyssey infrared imaging system (Li-COR Bioscience, United States).

### Mitochondrial morphology assessment

According to the manufacturer’s instructions, podocytes were cultured to 70% confluent and then incubated with 100 nM MitoTracker Red (Invitrogen, United States) in RPMI-1640 medium for 30 min. The confocal laser scanning microscope (LSM800, Zeiss, Germany) with the 63x/1.40 oil objective was applied to acquire mitochondrial fluorescence images at excitation/emission wavelengths of 578/598 nm. Mitochondrial average length and aspect ratio measurements were obtained using ImageJ software based on previously published methods (Ayanga et al., 2016). Aspect ratio was determined as the ratio between the major and minor axes of the ellipse equivalent to the mitochondrion. Quantification of mitochondrial average length and aspect ratio was calculated from five independent experiments (>100 mitochondria) for each group. Podocytes were fixed in 2.5% glutaraldehyde, and the ultrastructural changes were assessed by transmission electron microscopy (Hitachi, Japan).

### Mitochondrial membrane potential

Podocytes were treated with Mitochondrial Membrane Potential (MMP) detection reagent JC-1 (Beyotime, China), and then incubated for 15 min at 37°C according to the manufacturer’s protocols. JC-1 aggregates and JC-1 monomers respectively showed red and green fluorescence, which were detected by fluorescence microscopy (Axio Imager, Zeiss, Germany) with the 20x/0.7 objective. Fluorescence was measured at excitation/emission wavelengths of 480/590 nm (red) and 485/530 nm (green). The fluorescence intensity of MMP was analyzed semi-quantitatively using ImageJ software. Red and green fluorescence ratios in each experimental condition were measured from 15 random microscopy fields of three separate experiments for statistical analysis.

### Mitochondrial ROS determination

Mitochondrial superoxide was detected using the MitoSox Red Mitochondrial Superoxide Indicator (Invitrogen, United States) according to the manufacturer’s protocols. The fluorescence microscopy (Axio Imager, Zeiss, Germany) with the 20x/0.7 objective was applied to acquire fluorescence images at excitation/emission wavelengths of 480/590 nm. MitoSox Red fluorescence intensity in each experimental condition was measured from 15 random microscopy fields of three separate experiments using ImageJ software.

### TUNEL staining

TUNEL staining was performed using the *in situ* cell death detection kit (Roche, Switzerland) according to the manufacturer’s instructions. Podocytes were incubated with TUNEL reaction mixtures for 1 h and stained with DAPI for 5 min at 37°C in the dark, and then observed by fluorescence microscopy (Axio Imager, Zeiss, Germany) with 10x/0.45 objective at excitation/emission wavelengths of 485/530 nm (TUNEL) and 353/465 nm (DAPI). The percentage of apoptotic cells was measured through dividing apoptotic TUNEL-positive cell numbers by total cell numbers (more than 200 cells of five random fields in each experimental condition). Experiments were independently performed three times.

### Fluorescence measurement of intracellular Ca^2+^

Fluo-3/AM fluorescence-indicated Ca^2+^ entry was measured as described previously (Yang et al., 2013; Wang et al., 2019). Podocytes were cultured in physiological saline solution with Fluo-3/AM (Molecular Probes, United States) and Pluronic F-127 (Sigma, United States) for 45 min at 37°C. The cells were washed with Ca^2+^-free bath solution and treated with a variety of reagents as described in the results, including TG (1 μM), Ca^2+^ (CaCl_2_, 1.8 mM) and OAG (100 μM). Intracellular Ca^2+^ was detected by fluorescence using the laser scanning confocal microscope (FV300, Olympus, Japan) at excitation/emission wavelengths of 485/530 nm. The Ca^2+^ influx was presented as the ratio of the actual fluorescence intensity divided by the mean baseline fluorescence intensity. Data from 20 to 30 cells were averaged in a single run, and statistical analysis was performed on independent experiments with three batches of cell culture.

### CDK5 and calpain activity assays

CDK5/p25 kinase activity assay kit (Genmed, United States) was carried out according to the manufacturer’s protocols. The production of ADP was coupled to the oxidation of NADH using phosphoenolpyruvate (PEP) catalyzed by pyruvate kinase (PK) and lactic dehydrogenase (LDH). The cell lysate (100 μg) was incubated with the mixture including the PK/LDH system and the peptide substrate PKTPKKAKKL for 5 min at 30°C. The microplate reader (Molecular Devices, United States) was applied to examine the oxidation of NADH by measuring the absorbance at 340 nm. CDK5/p25 kinase activity was expressed as nmol NADH/min/mg of protein.

Calpain activity assay kit (Genmed, United States) was carried out according to the manufacturer’s protocols. The 200 μl mixture containing cell lysate (100 μg) was incubated for 60 min at 37°C with aminomethyl coumarin-labeled succinylated polypeptides, which is the substrate of calpain. Fluorescence produced by dissociative aminomethyl coumarin was detected by the fluorescence microplate reader (BioTek, United States) at excitation wavelength 380 nm and emission wavelength 460 nm.

### Statistical analysis

All experiments were conducted at least three times. Statistical analyses were performed using SPSS 25.0 software and graphs were prepared using GraphPad Prism 8. Data were expressed as mean ± standard deviation (SD). The statistical differences between two groups were analyzed by Student’s *t*-test (two-tailed). Comparisons of multiple groups were assessed using one-way ANOVA followed by Tukey’s *post-hoc* test. *p* < 0.05 indicated statistical significance.

## Results

### HG treatment increases TRPC6 expression levels and TRPC6-dependent Ca^2+^ influx in podocytes

**FIGURE 1:**
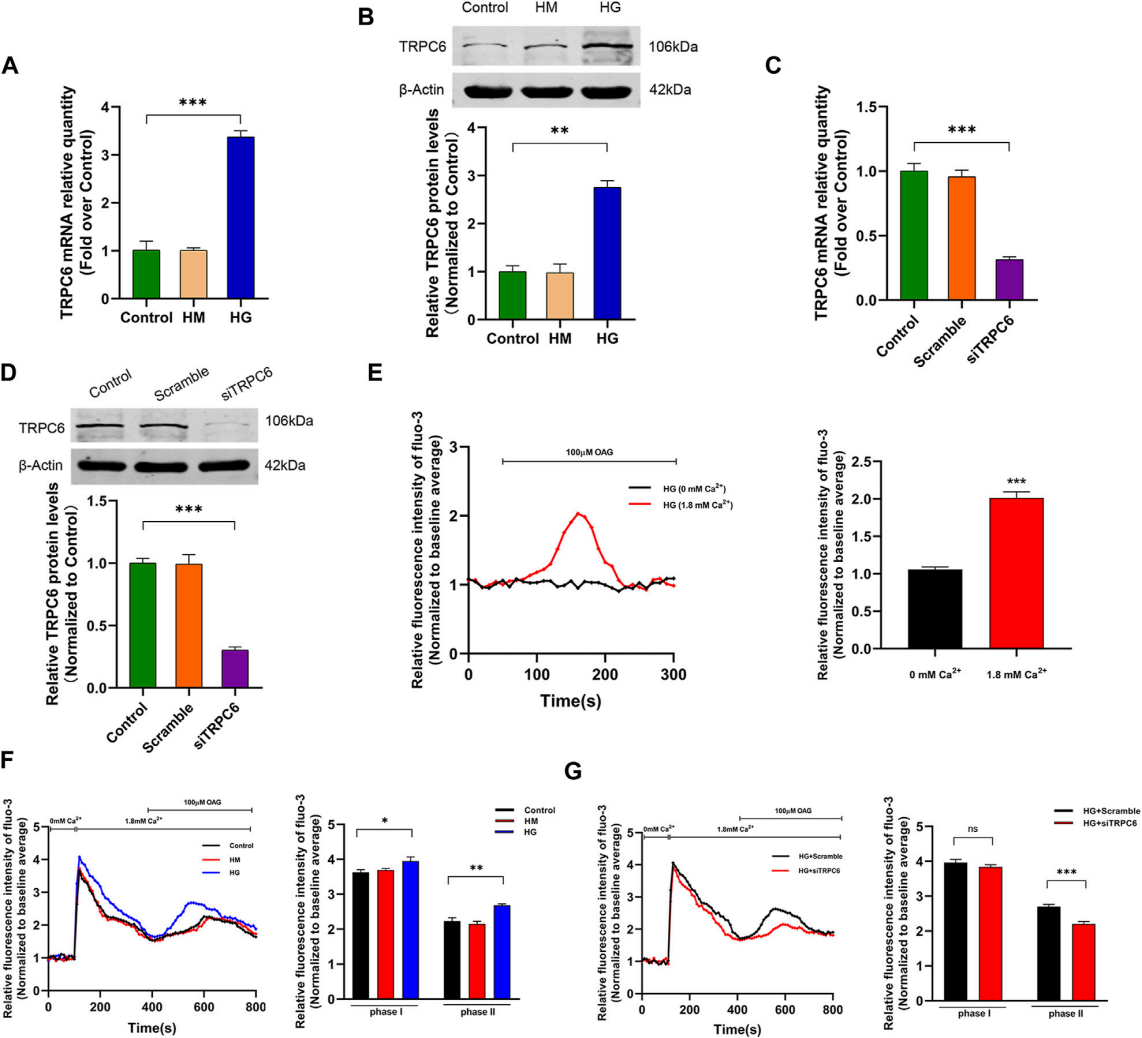
Effects of HG treatment on TRPC6 expression and TRPC6-dependent Ca^2+^ influx. **(A)** The mRNA expression levels of TRPC6 in the control, HM, and HG groups (*n* = 3 independent experiments). **(B)** Representative Western blot and quantitation analysis of TRPC6 in the control, HM, and HG groups (*n* = 3 independent experiments). **(C)** The mRNA expression levels of TRPC6 in the control, scramble, and siTRPC6 groups (*n* = 3 independent experiments). **(D)** Representative Western blot and quantitation analysis of TRPC6 in the control, scramble, and siTRPC6 groups (*n* = 3 independent experiments). **(E–G)** Confocal microscopy using Fluo-3 fluorescence methods was used to observe changes in the intracellular Ca^2+^ concentration. **(E)** Representative traces (left) and summary data (right) of the OAG-induced intracellular Ca^2+^ changes in the HG-treated podocytes, incubated with or without 1.8 mM Ca^2+^ (*n* = 3 independent experiments). **(F)** Representative traces (left) and summary data (right) of the TG-induced SOCE (phase Ⅰ) and OAG-induced ROCE (phase Ⅱ) in the control, HM, and HG groups (*n* = 3 independent experiments). **(G)** Representative traces (left) and summary data (right) of the TG-induced SOCE (phase Ⅰ) and OAG-induced ROCE (phase Ⅱ) in the HG + scramble and HG + siTRPC6 groups (*n* = 3 independent experiments) ns, no statistical significance. *p < 0.05; **p < 0.01; ***p < 0.001.

We first investigated the effect of HG treatment on TRPC6 expression in cultured human podocytes by PCR and Western blot analysis. 30 mM glucose treatment for 48 h significantly increased TRPC6 mRNA and protein expression levels, and there was no difference between 30 mM mannitol-treated podocytes for 48 h and control podocytes (Figures 1A,B). This result was consistent with our previous study (Yang et al., 2013). Real-time PCR and Western blot data revealed that transfection by TRPC6 siRNA effectively reduced TRPC6 expression in podocytes (Figures 1C,D).

To evaluate the TRPC6-induced Ca^2+^ influx in HG-treated podocytes, we detected intracellular Ca^2+^ fluorescence intensity. As shown in Figure 1E, Podocytes were treated with 30 mM glucose for 48 h. The addition of OAG (a diacylglycerol analogue) in extracellular solution with 1.8 mM Ca^2+^ significantly increased Ca^2+^ influx in podocytes, while OAG failed to change intracellular Ca^2+^ in calcium-free solution, indicating that extracellular Ca^2+^ influx led to OAG-induced Ca^2+^ influx peak in HG-induced podocytes. To examine the effect of HG treatment on the intracellular calcium homeostasis in podocytes and the role of TRPC6 in the progress, we first treated podocytes with 1 μM TG for 5 min in calcium-free solution to depleting intracellular Ca^2+^ stores (Figures 1F,G). The TG-induced store-operated Ca^2+^ entry (SOCE) was quantified as the rapid elevation in Ca^2+^ influx (phase Ⅰ) when 1.8 mM Ca^2+^ was re-added to the bath solution after depletion of stored calcium with TG. Subsequently, OAG-induced receptor-operated Ca^2+^ entry (ROCE) showed an additional and significant increase of intracellular Ca^2+^ (phase Ⅱ) under the condition where Ca^2+^ stores were already depleted. Compared to control, HG treatment both increased TG-induced SOCE and OAG-induced ROCE, and the latter showed a more statistically significant difference (Figure 1F). Transfection with TRPC6 siRNA significantly decreased OAG-mediated Ca^2+^ entry compared to scramble siRNA. While TG-induced SOCE was unaffected in podocytes under HG conditions (Figure 1G). Taken together, these results showed that HG-induced ROCE was dependent on the TRPC6 channel in podocytes.

### TRPC6 inhibition attenuates HG-induced mitochondrial dysfunction and podocyte apoptosis

**FIGURE 2:**
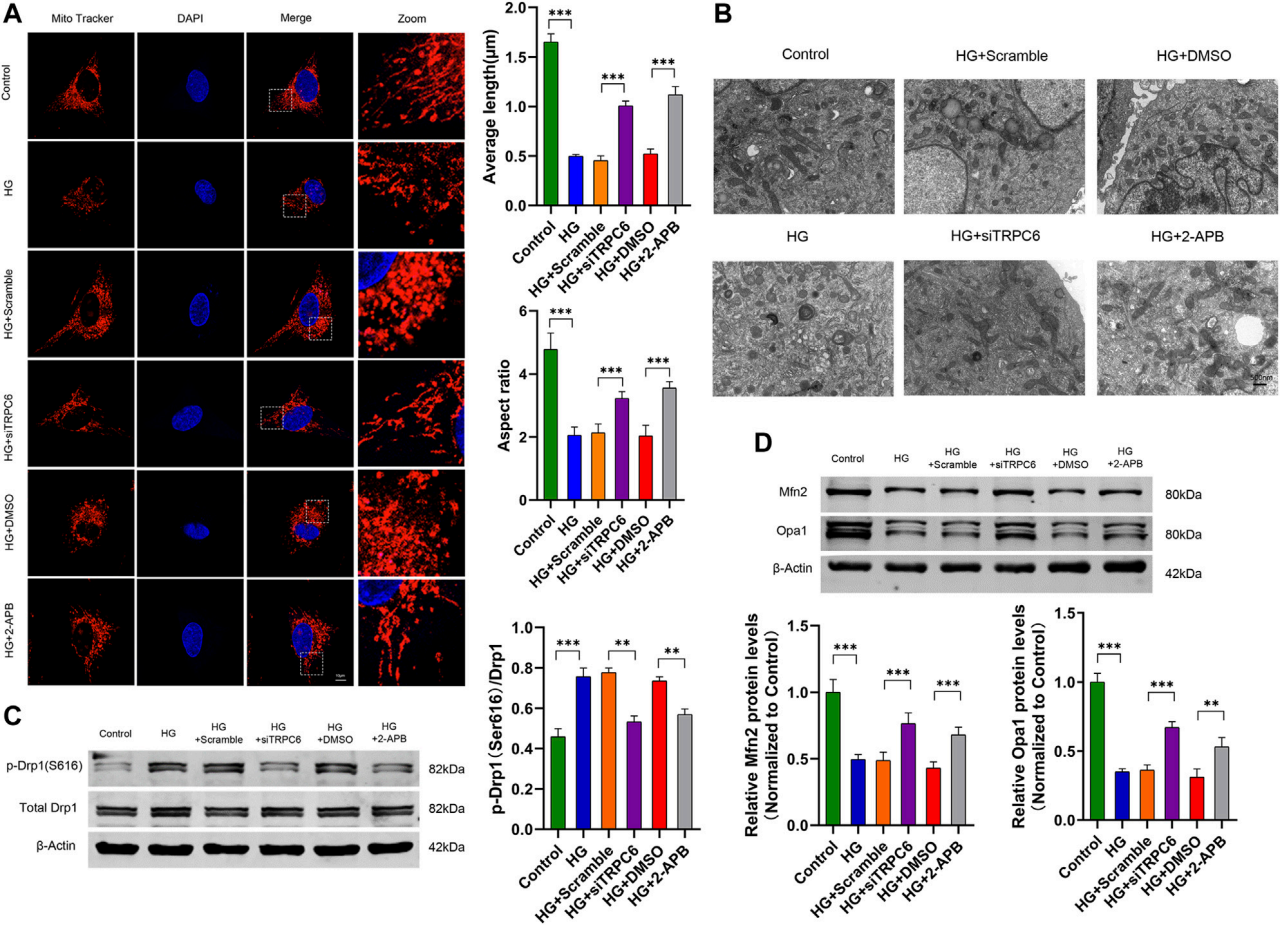
Effects of TRPC6 inhibition on mitochondrial morphology in podocytes under HG conditions. **(A)** MitoTracker Red and DAPI staining, and analysis of average length and aspect ratio by ImageJ software in the control, HG, HG + scramble, HG + siTRPC6, HG + DMSO and HG + 2-APB groups. Scale bars, 10 μm (*n* = 5 independent experiments). **(B)** Representative electron micrographs in the six groups. Scale bars, 500 nm. **(C)** Representative Western blot and quantitation analysis of p-Drp1 (Ser616) and total Drp1 in the six groups (*n* = 3 independent experiments). **(D)** Representative Western blot and quantitation analysis of Mfn2 and Opa1 in the six groups (*n* = 3 independent experiments). **p < 0.01; ***p < 0.001.

To investigate the effects of TRPC6 inhibition on HG-induced mitochondrial morphological and functional changes in cultured human podocytes, we transfected podocytes with TRPC6 siRNA or treated them with 2-APB (a pharmacological inhibitor of TRPC6). MitoTracker Red staining and electron microscopy showed punctate and fragmented mitochondria in the HG group podocytes (30 mM glucose for 48 h), whereas long filamentous mitochondria were observed in the siTRPC6 group and 2-APB group under HG conditions (Figures 2A,B). Compared to the control group, the average length and aspect ratio of mitochondria decreased in the HG group, which was attenuated in the siTRPC6 group and 2-APB group under HG conditions (Figure 2A). Meanwhile, we found that HG increased the phosphorylation levels of Drp1 at S616, and decreased mitochondrial fusion protein Mfn2 and Opa1 levels. Compared to the HG group, podocytes after siTRPC6 transfection or 2-APB treatment resulted in decreased expression levels of p-Drp1(S616) and increased expression levels of Mfn2 and Opa1 (Figures 2C,D). Results showed that TRPC6 inhibition alleviated HG-induced mitochondrial fission *via* downregulating Drp1 phosphorylation at the S616 site.

**FIGURE 3:**
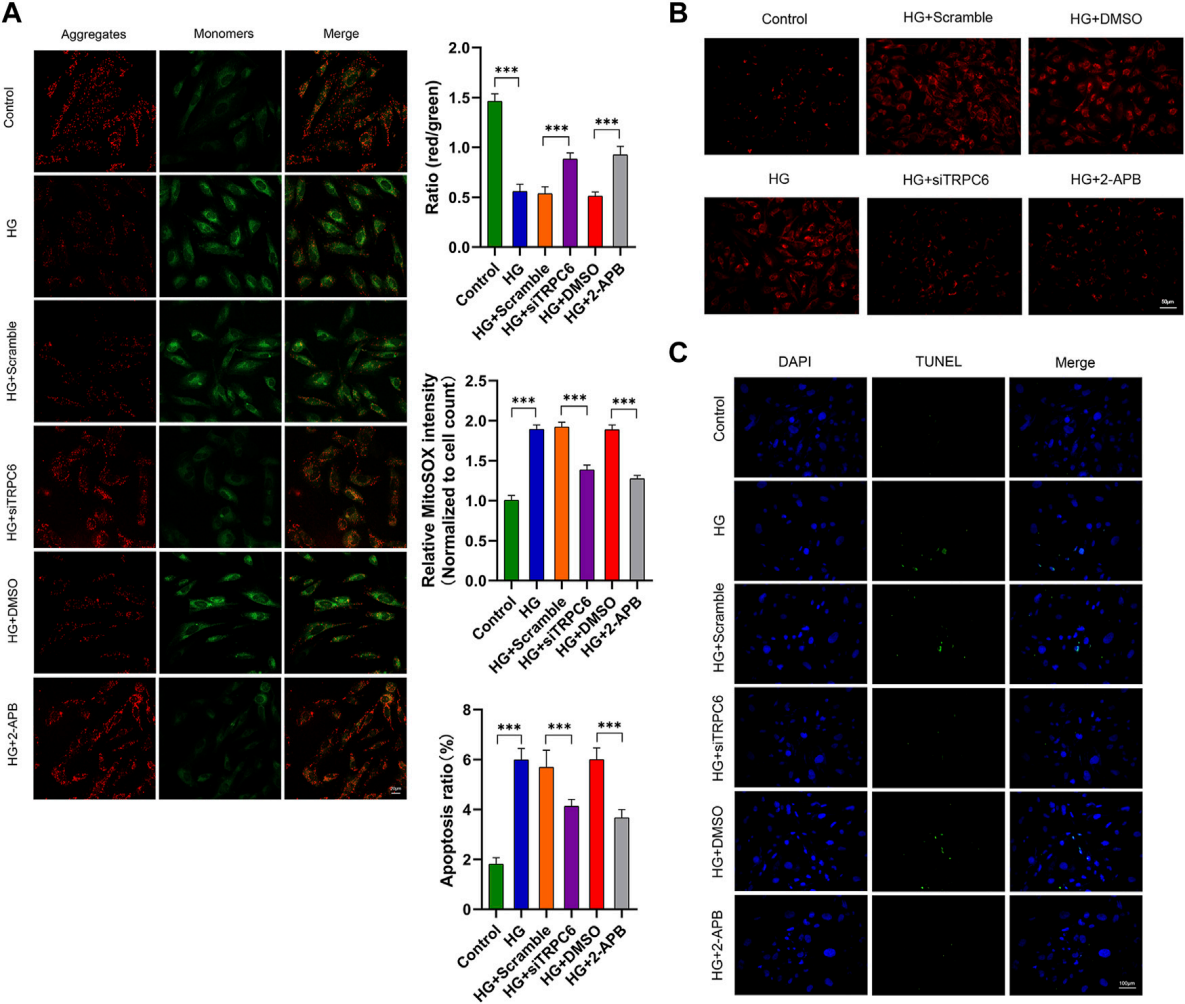
Effects of TRPC6 inhibition on mitochondrial functions in podocytes under HG conditions. **(A)** JC-1 staining and fluorescence analysis in the control, HG, HG + scramble, HG + siTRPC6, HG + DMSO, and HG + 2-APB groups. Scale bars, 20 μm (*n* = 3 independent experiments). **(B)** MitoSOX Red staining and fluorescence analysis in the six groups. Scale bars, 50 μm (*n* = 3 independent experiments). **(C)** TUNEL staining and quantitation analysis of apoptotic cell number (%) in the six groups. Scale bars, 100 μm (*n* = 3 independent experiments). ***p < 0.001.

To further identify the effect of TRPC6 on HG-induced mitochondrial dysfunction, we measured mitochondrial membrane potential, mitochondrial ROS and podocyte apoptosis respectively by staining with JC-1, MitoSOX and TUNEL. The results exhibited that HG treatment decreased MMP, increased mtROS and promoted apoptosis, all of which can be reversed by siTRPC6 transfection or 2-APB treatment (Figures 3A–C). These results suggested that TRPC6 activation played an important role in HG-induced mitochondrial dysfunction and podocyte apoptosis.

### BAPTA-AM alleviates HG-induced mitochondrial fission

**FIGURE 4:**
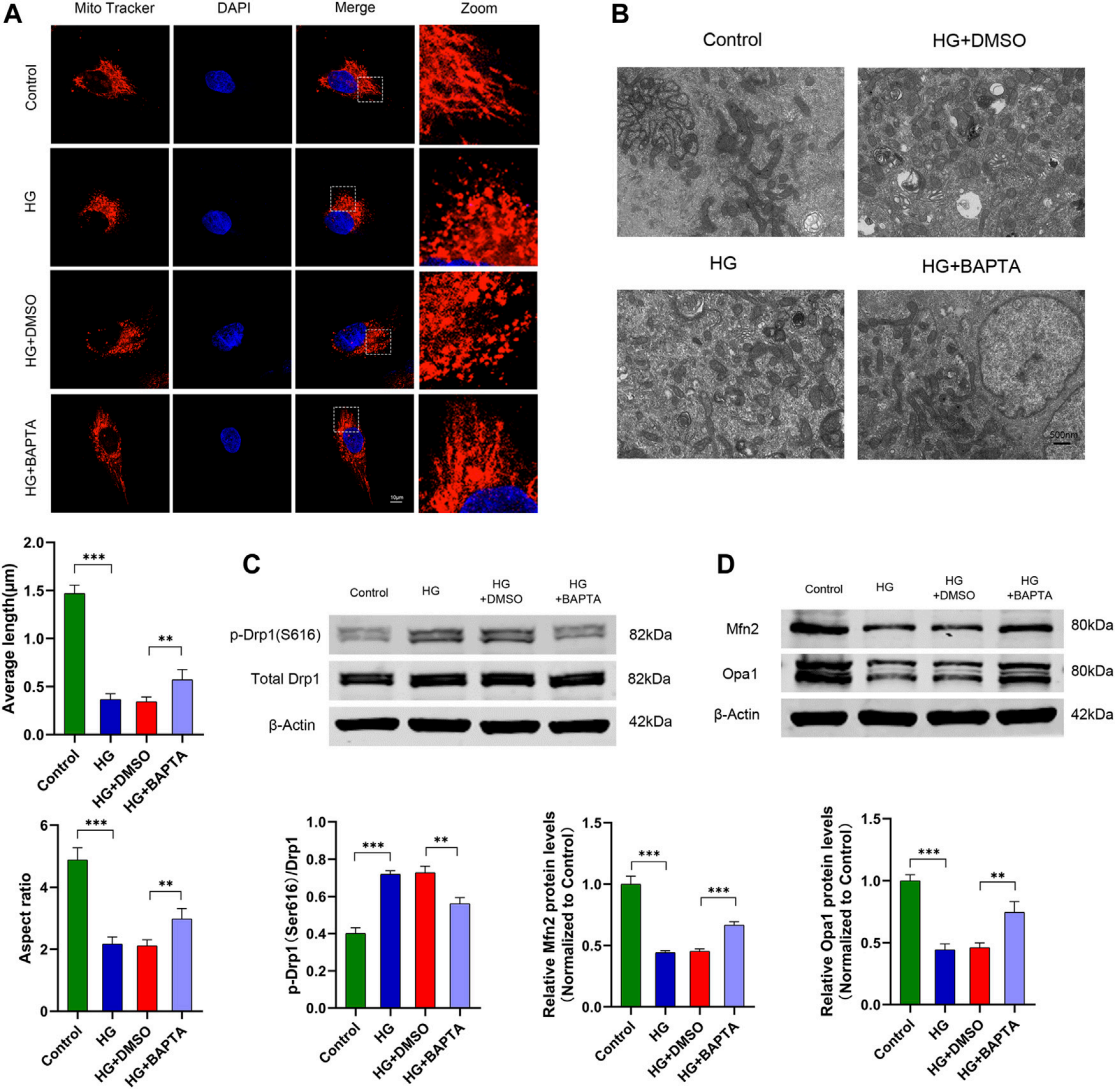
BAPTA-AM alleviates HG-induced mitochondrial fission in podocytes. **(A)** MitoTracker Red and DAPI staining, and analysis of average length and aspect ratio by ImageJ software in the control, HG, HG + DMSO and HG + BAPTA groups. Scale bars, 10 μm (*n* = 5 independent experiments). **(B)** Representative electron micrographs in the four groups. Scale bars, 500 nm. **(C)** Representative Western blot and quantitation analysis of p-Drp1 (Ser616) and total Drp1 in the four groups (*n* = 3 independent experiments). **(D)** Representative Western blot and quantitation analysis of Mfn2 and Opa1 in the four groups (*n* = 3 independent experiments). **p < 0.01; ***p < 0.001.

Increasing evidence indicates that intracellular Ca^2+^ dynamically regulates mitochondrial fission in various cell types under HG conditions (Hom et al., 2007; Hom et al., 2010; Wu et al., 2021). To verify that in cultured human podocytes, we treated podocytes with BAPTA-AM (an intracellular calcium chelating agent) in HG conditions. The mitochondrial morphology was examined by MitoTracker Red staining and electron microscopy, which exhibited that HG-induced mitochondrial fragmentation was prevented by the BAPTA-AM treatment (Figures 4A,B). As Figure 4A shows, the quantification of mitochondrial average length and aspect ratio increased in podocytes treated with BAPTA-AM following the HG stimulation, compared to the HG group. Additionally, we found that BAPTA-AM resulted in the expression levels decreasing for p-Drp1(S616) while increasing for Mfn2 and Opa1 in HG-treated podocytes, separately (Figures 4C,D). These data suggested that the blockage of intracellular Ca^2+^ inhibited HG-induced mitochondrial fission in podocytes.

### TRPC6 mediates calpain-1 activation to regulate HG-induced mitochondrial fission

**FIGURE 5:**
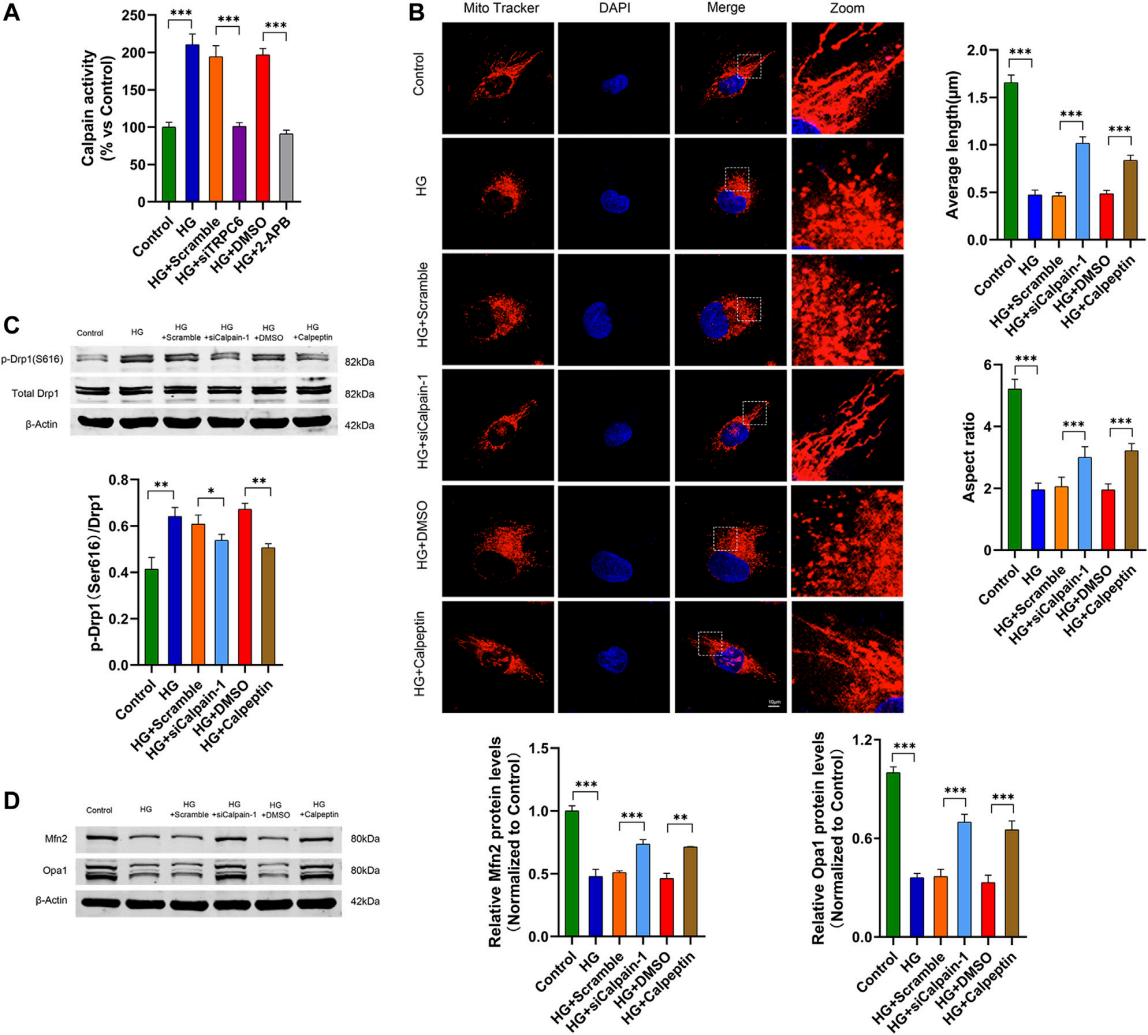
TRPC6 mediates HG-induced mitochondrial fission through calpain-1 activation in podocytes. **(A)** Calpain activity assay data in the control, HG, HG + scramble, HG + siTRPC6, HG + DMSO, and HG + 2-APB groups (*n* = 5 independent experiments). **(B)** MitoTracker Red and DAPI staining, and analysis of average length and aspect ratio by ImageJ software in the control, HG, HG + scramble, HG + siCalpain-1, HG + DMSO, and HG + calpeptin groups. Scale bars, 10 μm (*n* = 5 independent experiments). **(C)** Representative Western blot and quantitation analysis of p-Drp1 (Ser616) and total Drp1 in the six groups (*n* = 3 independent experiments). **(D)** Representative Western blot and quantitation analysis of Mfn2 and Opa1 in the six groups (*n* = 3 independent experiments). *p < 0.05; **p < 0.01; ***p < 0.001.

Multiple studies have demonstrated that TRPC6-mediated Ca^2+^ influx activates cytoplasmic calpain-1 in podocytes (Verheijden et al., 2018; Ding et al., 2021). To investigate the effect of calpain-1 on TRPC6 mediating mitochondrial fission in HG-stimulated podocytes, we first measured the activity of calpain in podocytes respectively transfected with siTRPC6 and treated with 2-APB in HG conditions, and found that TRPC6 inhibition decreased the activity of calpain in HG-treated podocytes (Figure 5A). These results indicated that calpain-1 may be a downstream effector of TRPC6 in podocytes. Subsequently, we evaluated HG-induced mitochondrial fission after calpain-1 inhibition. Podocytes treated with HG showed small, round mitochondria by MitoTracker Red staining. While in comparison, podocytes transfected with calpain-1 siRNA or treated with calpeptin under HG conditions showed decreased mitochondrial fragmentation, as evidenced by an increase in typical tubular and long filamentous mitochondria, consistent with the quantification of the mitochondrial average length and aspect ratio (Figure 5B). At the same time, western blot analysis of mitochondrial dynamic proteins exhibited that HG increased the expression levels of p-Drp1(S616) and decreased the levels of Mfn2 and Opa1. Knockdown calpain-1 expression or treatment with calpeptin attenuated all these changes caused by HG (Figures 5C,D). These findings indicated that calpain-1 was an important downstream effector of the TRPC6 channel on mitochondrial fission in HG-treated podocytes.

### TRPC6 activates CDK5 through the Ca^2+^/calpain-1 pathway in HG-treated podocytes

**FIGURE 6:**
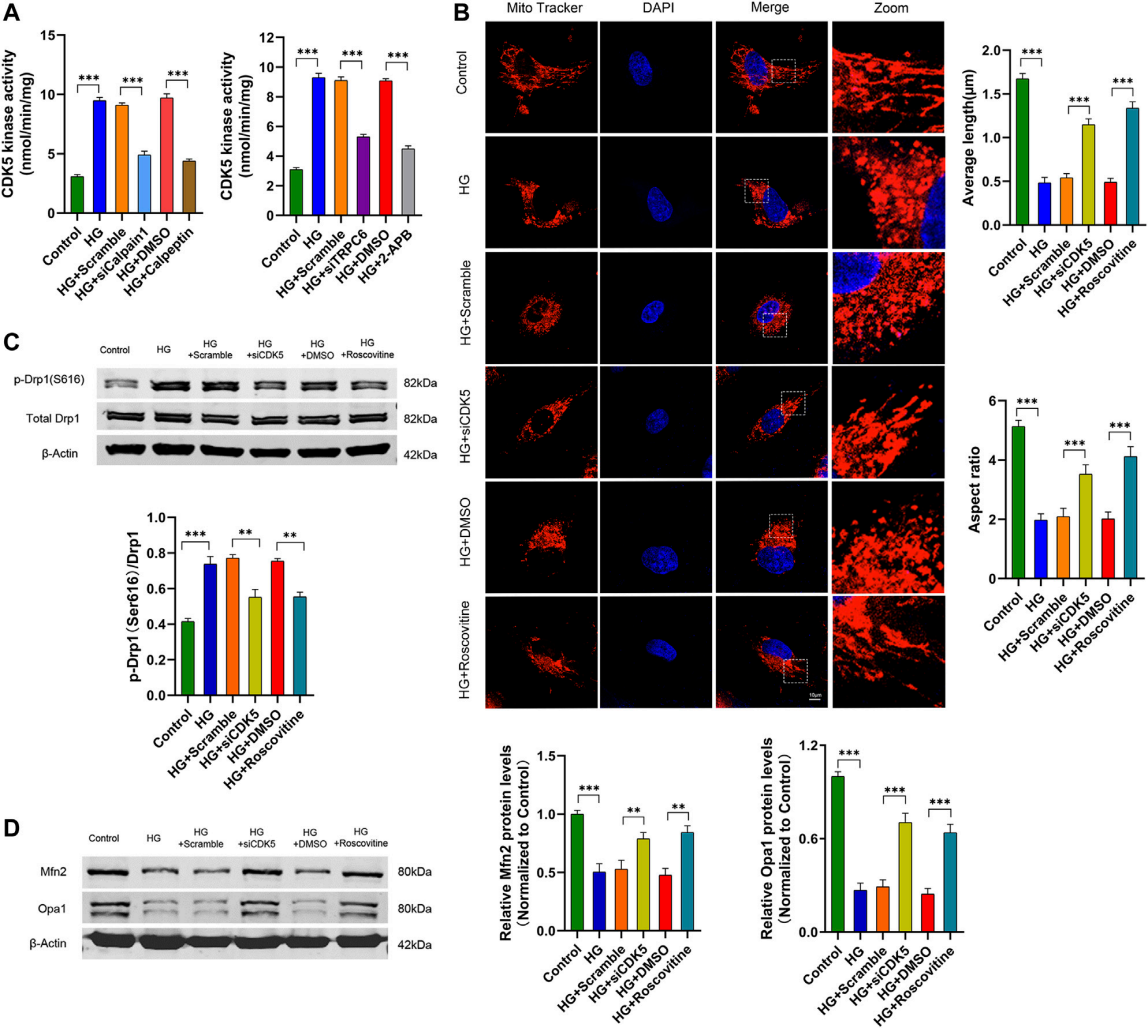
TRPC6 activates CDK5 through the Ca^2+^/calpain-1 pathway which contributes to HG-induced mitochondrial fission in podocytes. **(A)** CDK5 kinase activity assay data in the control, HG, HG + scramble, HG + siCalpain-1, HG + DMSO and HG + Calpeptin groups (left), and in the control, HG, HG + scramble, HG + siTRPC6, HG + DMSO and HG + 2-APB groups (right) (*n* = 5 independent experiments). **(B)** MitoTracker Red and DAPI staining, and analysis of average length and aspect ratio by ImageJ software in the control, HG, HG + scramble, HG + siCDK5, HG + DMSO, and HG + roscovitine groups. Scale bars, 10 μm (*n* = 5 independent experiments). **(C)** Representative Western blot and quantitation analysis of p-Drp1 (Ser616) and total Drp1 in the six groups (*n* = 3 independent experiments). **(D)** Representative Western blot and quantitation analysis of Mfn2 and Opa1 in the six groups (*n* = 3 independent experiments). **p < 0.01; ***p < 0.001.

Studies have shown that calpain-1 increased CDK5 activity by regulating its co-activator, namely converting p35 to effective p25 in cultured neurons (Kusakawa et al., 2000; Park et al., 2017). Hence, we considered whether calpain-1 mediated HG-induced mitochondrial fission by increasing CDK5 kinase activity in cultured human podocytes. As shown in Figure 6A, both knockdown of calpain-1 with siRNA and pharmacological inhibition of calpain-1 with calpeptin downregulated CDK5 kinase activity, indicating that calpain-1 was an upstream regulator of CDK5 in podocytes under HG conditions. Simultaneously, we measured CDK5 kinase activity in podocytes after TRPC6 inhibition. Results showed that podocytes transfected with siTRPC6 or treated with 2-APB reduced CDK5 kinase activity under HG conditions (Figure 6A). These results indicated that CDK5 was an important downstream regulator of TRPC6.

To confirm the effect of CDK5 on HG-induced mitochondrial fission in cultured human podocytes, we evaluated mitochondrial fission by MitoTracker Red staining and Western blot. Confocal images showed that compared to short and round mitochondria in HG-treated podocytes, mitochondria in HG-stimulated podocytes transfected by siCDK5 or treated with roscovitine (a CDK5 inhibitor) formed long filaments and increased the average length and aspect ratio (Figure 6B). Additionally, Western blot analysis of mitochondrial morphology-related proteins exhibited decreased fission-related protein p-Drp1 (S616) levels and simultaneously increased fusion-related protein Mfn2 and Opa1 levels in CDK5-inhibited podocytes under HG conditions, compared with HG treated podocytes (Figures 6C,D). These results verified that CDK5 mediated HG-induced mitochondrial fission in cultured human podocytes. Collectively, TRPC6 activated CDK5 through the Ca^2+^/calpain-1 pathway, contributing to HG-induced mitochondrial fission in human podocytes.

## Discussion

Abnormal mitochondrial dynamics are involved in the occurrence and development of DN (Saxena et al., 2019; Audzeyenka et al., 2022). The role of TRPC6 in the progression of DN is still not completely understood. In this study, we found that TRPC6 inhibition attenuated HG-induced mitochondrial fission, ROS production and cellular apoptosis in podocytes. We confirmed that TRPC6 regulated the activation of CDK5 through the Ca^2+^/calpain-1 pathway to mediate mitochondrial fission in HG-stimulated podocytes. To our knowledge, this is the first study to report that TRPC6 plays an important role in HG-induced mitochondrial fission in cultured human podocytes.

Disturbance of intracellular Ca^2+^ homeostasis is a major cause of podocyte structural and functional injury. Studies have shown that intracellular Ca^2+^ regulates mitochondrial dynamics and ROS generation in cardiomyocytes and liver cells (Hom et al., 2010; Surmeier et al., 2017; Jhun et al., 2018). Consistent with previous studies, we found that intracellular Ca^2+^ inhibition by BAPTA treatment attenuates mitochondrial fission in HG-stimulated podocytes. In the study, we focused on TRPC6, a Ca^2+^-permeable non-selective cation channel, which is strongly associated with glomerular diseases. We found that HG treatment not only increased TRPC6 expression levels but also enhanced TRPC6-dependent Ca^2+^ influx in podocytes. Furthermore, the effect of TRPC6 on Drp1-mediated mitochondrial fission was described in the hippocampus of rats (Ko and Kang, 2017), which remains to be explored in podocytes. Our results are consistent with those reports and reveal that the inhibition of TRPC6 by 2-APB or siRNA significantly attenuates HG-induced mitochondrial fission.

Phosphorylation of Drp1 is one of the vital posttranslational protein modifications, which regulates its recruitment to the mitochondria (Schmitt et al., 2018; Rong et al., 2020; Kraus et al., 2021). Drp1 phosphorylation at S616 and S637 was extensively reported to play a critical regulatory role on mitochondrial fission, and that may cause different effects in various cell types or disease models (Chang and Blackstone, 2010; Ayanga et al., 2016; Bo et al., 2018; Feng et al., 2020). A previous study suggested that the increase of Drp1 phosphorylation at S616 activated by CDK5 enhanced mitochondrial fission and DN progression (Wang et al., 2021). Additionally, another study indicated that ROCK1-mediated Drp1 phosphorylation contributed to excessive mitochondrial fission by translocating Drp1 to the mitochondria (Wang et al., 2012). Consistent with those results, we found that HG-induced mitochondrial fission by simultaneously upregulating the phosphorylation of Drp1 at S616 and downregulating the expression levels of Mfn2 and Opa1. In response to the increase of fission and decrease of fusion, mitochondrial damages on morphology and function contribute to ROS overproduction and mitochondrial apoptotic pathway activation, resulting in podocytes apoptosis (Wang et al., 2012; Zorov et al., 2014; Fan et al., 2019; Chan, 2020; Chen et al., 2020). In this study, we found that the inhibition of TRPC6 alleviated HG-induced mitochondrial fragmentation, MMP decrease, ROS overproduction and extensive cellular apoptosis in podocytes.

Calpain-1, activated by Ca^2+^ influx, is a key downstream target of TRPC6 (Zhang et al., 2014; Verheijden et al., 2018). Previous studies have shown that the activation of calpain induces mitochondrial fission by mediating Drp1 phosphorylation, resulting in myocardial apoptosis (Shi et al., 2021; Zhang et al., 2021). Studies have identified that inhibition of either calpain or calcineurin leads to downregulated Drp1 and Fis1 levels, and upregulated Opa1 levels and MMP during exposure to oxidative stress. Such an effect prevents neurons from mitochondrial dynamics impairment (Tangmansakulchai et al., 2016). However, it is still unknown about the effect of calpain-1 in HG-induced podocyte mitochondrial fission. Consistent with previous reports, we found that calpain activity was upregulated in podocytes under hyperglycemia, which was abolished by TRPC6 inhibition. In addition, both the pharmacological blockage and the knockdown of calpain-1 alleviated mitochondrial fission induced by HG in podocytes. Accordingly, the results indicate that calpain-1 regulates mitochondrial dynamics impairment as the downstream regulator of TRPC6 in podocytes under HG conditions.

The overactivation of CDK5 induced by HG is tightly responsible for podocyte injury in diabetes (Lee et al., 2000; Liu et al., 2012; Zheng et al., 2016; Zhang et al., 2017; Wang et al., 2021). Consistent with previous reports, we found that CDK5 inhibition by siRNA or roscovitine alleviated HG-induced mitochondrial fragmentation by increasing Drp1 phosphorylation at the S616 site in podocytes. Studies mainly focusing on the nervous system suggest that Ca^2+^/calpain activates CDK5 *via* cleavage p35 to p25, which is a highly active molecular chaperone (Lee et al., 2000; Shu et al., 2018). We measured CDK5/p25 activity in this study since the complex performs strong and stable activities, and found that HG treatment increased CDK5 kinase activity, whereas the effect could be abolished by calpain-1 or TRPC6 inhibition. These results indicate that TRPC6 activates CDK5 in a Ca^2+^/calpain-1 dependent manner, contributing to mitochondrial fission in HG-treated podocytes.

In this study, we provide insight into the effect of TRPC6 on mitochondrial injury in diabetic podocyte models. Firstly, we demonstrate that TRPC6 plays an important role in the progression of DN by regulating mitochondrial dynamics. Secondly, we confirm that the intracellular Ca^2+^ chelator alleviates mitochondrial fission in podocytes. And finally, we identify TRPC6 as a critical upstream regulator of CDK5 activation in modulating HG-induced mitochondrial fission through the Ca^2+^/calpain-1 pathway in podocytes.

A potential limitation of the study is that the involvement of other channels/signaling in HG-induced mitochondrial fission in podocytes is not fully excluded. Although we showed the importance of TRPC6-dependent Ca^2+^ influx in regulating mitochondrial dynamics, TRPC3/7 channel with similar structures activated by OAG may also play a role. Moreover, 2-APB treatment non-selectively blocked TRPCs and store-operated Ca^2+^ channels, and intracellular Ca^2+^ measurements showed that HG treatment also increased TG-induced SOCE, indicating that Orai-STIM dependent Ca^2+^ influx may be involved in HG-induced mitochondrial fission in podocytes. Since roscovitine was a selective inhibitor that substantially inhibited CDK5 and CDK2, we do not exclude that CDK2 may also play a role in the TRPC6-mediated mitochondrial fission in HG-treated podocytes. The expression of calpain-2 has been reported in podocytes, but whether it is involved in HG-mediated mitochondrial fission as the downstream regulator of TRPC6 deserves additional exploration in future studies. In the study, we detected MMP, mitochondrial ROS and podocyte apoptosis by respectively staining podocytes with JC-1, MitoSOX and TUNEL, and these measurement methods also have limitations in quantitative accuracy.

In conclusion, our findings uncover that TRPC6 mediates HG-induced mitochondrial fission in podocytes through the Ca^2+^/calpain-1/CDK5 pathway. Our results suggest that the TRPC6/Ca^2+^/calpain-1/CDK5 signal pathway can be a promising therapeutic target for DN.

## Data Availability

The original contributions presented in the study are included in the article/supplementary material, further inquiries can be directed to the corresponding author.

## Abbreviations

AMPK, Adenosine monophosphate-activated protein kinase; 2-APB, 2-aminoethoxydiphenyl borate; CDK2, cyclin-dependent kinase 2; CDK5, cyclin-dependent kinase 5; DN, diabetic nephropathy; Drp1, dynamin-related protein 1; HG, high glucose; LDH, lactic dehydrogenase; Mfn1, mitofusin 1; Mfn2, mitofusin 2; MMP, mitochondrial membrane potential; OAG, 1-oleoyl-2-acetyl-glycerol; Opa1, optic atrophy 1; PEP, phosphoenolpyruvate; PK, pyruvate kinase; ROCE, receptor-operated Ca^2+^ entry; ROS, reactive oxygen species; SOCE, store-operated Ca^2+^ entry; TRPC6, transient receptor potential canonical channel-6.
